# Chronic Political Instability and the HIV/AIDS Response in Guinea-Bissau from 2000 to 2015: A Systematic Review

**DOI:** 10.3390/tropicalmed6010036

**Published:** 2021-03-16

**Authors:** Joshua Galjour, Philip Havik, Peter Aaby, Amabelia Rodrigues, Emmanuel Kabengele Mpinga

**Affiliations:** 1 Institute of Global Health, University of Geneva, 1202 Geneva, Switzerland; Emmanuel.Kabengele@unige.ch; 2 Centre for Global Health and Tropical Medicine (GHTM), Institute of Hygiene and Tropical Medicine, Universidade NOVA de Lisboa, 1349-008 Lisbon, Portugal; philip.havik@ihmt.unl.pt; 3 Bandim Health Project, Apartado 8611004, Bissau Codex, Guinea-Bissau; p.aaby@bandim.org (P.A.); a.rodrigues@bandim.org (A.R.)

**Keywords:** Guinea-Bissau, West Africa, HIV/AIDS, governance, political stability, political epidemiology

## Abstract

Guinea-Bissau suffers from political instability and an unusually high HIV/AIDS burden compared to other countries in the West Africa region. We conducted a systematic review on the HIV/AIDS epidemic in Guinea-Bissau during the Millennium Development Goals (MDGs) period (2000–2015), which dovetailed with a period of chronic political instability in the country’s history. We searched published works on the HIV/AIDS epidemic in Guinea-Bissau for references to chronic political instability. Six databases and the grey literature were searched, informed by expert opinion and manual research through reference tracing. Preferred Reporting Items for Systematic Reviews and Meta-Analyses (PRISMA) guidelines were followed. The search yielded 122 articles about HIV/AIDS in Guinea-Bissau during the MDG years. Biomedical, clinical, or epidemiological research predominated public health research production on HIV/AIDS in Guinea-Bissau in this period. Six articles addressing themes related to chronic political instability, including how political instability has affected the HIV/AIDS disease response, were identified. The results suggest the importance of considering a broader political epidemiology that accounts for socio-political aspects such as governance, human rights, and community responses into which any national HIV/AIDS response is integrated.

## 1. Introduction

Significant progress has been achieved in the global fight against HIV/AIDS in the last twenty years [1]. Despite these results, which have led to HIV/AIDS becoming a chronically manageable illness over time (distinct from its “framing as a human emergency” [2]), the disease as a social pathology continues to influence, and is influenced by a country’s larger social, economic, and political context. The level of political stability seems to affect a country’s response to HIV/AIDS [3,4,5]. A recent comparative survey of 137 countries established a correlation between countries with lower levels of peace and higher corruption levels and countries with lower antiretroviral therapy (ART) coverage (*p* < 0.001), suggesting “that there may be a threshold of conflict and corruption levels, below which ART coverage is particularly compromised” [6].

The case of Guinea-Bissau, a small country in West Africa, provides an opportunity to assess how political instability may have affected its unique HIV/AIDS epidemic. Guinea-Bissau suffers from political instability and an unusually high HIV/AIDS burden. While HIV/AIDS remains, to varying degrees, a public health threat in the neighboring countries of The Gambia, Senegal and Guinea, the data on HIV incidence and prevalence suggest that Guinea-Bissau has been significantly more affected than neighboring countries. Appendix A presents the HIV/AIDS incidence and prevalence for the four countries. During the same period (2000–2015), the neighboring countries of Senegal, The Gambia, and Guinea all remained relatively stable or experienced more isolated periods of political instability or uncertainty. 

In contrast, Guinea-Bissau emerged from a protracted war of liberation (1963–1974) with its colonizer Portugal and has experienced periods of political instability throughout its existence, as well as violent episodes such as a civil war (1998–1999) [7]. Guinea-Bissau’s cycles of political instability have left it in a “fragility trap” as “one of the most politically unstable countries in the world,” according to the International Monetary Fund [8]. Appendix B provides a timeline of the major milestones in the nation’s history during this 15-year period. Government instability has been defined as “unforeseen and unexpected events such as the end of a government or of an electorate that occurs either legally or by force” [9]. We pragmatically define “chronic political instability” as the lack of functioning government institutions in line with the constitution, laws, and non-written governing traditions of a nation, as well as the manifestations it most commonly takes, such as through civil war, frequent regime change, assassinations, and attempted and successful coups d’état. If, as the World Bank has put forward, “good governance in health systems promotes effective delivery of health services,” then it would be reasonable to assume that the chronicity of Guinea-Bissau’s instability during its post-civil war period undermined the provision of health services, including those related to HIV/AIDS [10].

Beginning around 2003, many countries in sub-Saharan Africa including Guinea-Bissau began to scale up national responses to HIV/AIDS as external funding became more widely available. Following the civil war (1998–1999), the period of the Millennium Development Goals (MDGs) (2000–2015) coincided with a period of chronic political instability in the country. Our hypothesis was that political, administrative, and institutional factors could have hindered the scale-up of HIV/AIDS services and may have increased HIV/AIDS-related social and economic vulnerabilities. While the HIV/AIDS epidemic in Guinea-Bissau and the nature of the country’s political instability have both been investigated, we are not aware of any comprehensive review of the intersection of these two research domains.

To fill this gap, we aimed to systematically review the published data on HIV/AIDS in Guinea-Bissau, explore the role of political instability in the literature on the country’s HIV/AIDS epidemic, and highlight areas that might be considered in future research.

## 2. Methods

### 2.1. Literature Search

The search objective was to survey the HIV/AIDS knowledge base related to the MDG period of 2000–2015 in Guinea-Bissau and to identify the effects of political instability on HIV outcomes. The literature review was based on the following three domains: (i.) an electronic search of the peer-reviewed literature; (ii.) grey literature, informed by expert opinion; and (iii.) manual research through reference tracing of both i. and ii. The following six electronic databases were used to identify articles in the peer-reviewed literature: PubMed, Web of Science, SciELo, Embase, Banque de Données en Santé Publique (BDSP), and Lilac. The electronic search was conducted in 2017, and the search included records about Guinea-Bissau written in English, French, and Portuguese. Portuguese was included because it is the country’s official language, and French was included because the country’s geography is surrounded by French-speaking African countries and the fact that French is sometimes spoken in the country. The search terms included “Guinea-Bissau,” “HIV/AIDS,” “public health programs,” and “political instability.” Search-engine specific variations of the search terms were used. Translations of search words in Portuguese and French were used where appropriate. 

### 2.2. Inclusion Criteria

The following items were included: original articles, literature reviews, and scholarly works (reports, books, theses, and dissertations). The following items were excluded through manual screening: papers without an abstract, official recommendations, judicial comments or decisions, declarations, conference proceedings, book reviews, press releases, and glossaries. Works in other languages besides English, French, and Portuguese were excluded, as were works published prior to 1 January 2000 or works that did not relate to the 15-year period under review (2000–2015). 

### 2.3. Data Analysis

**Figure 1:**
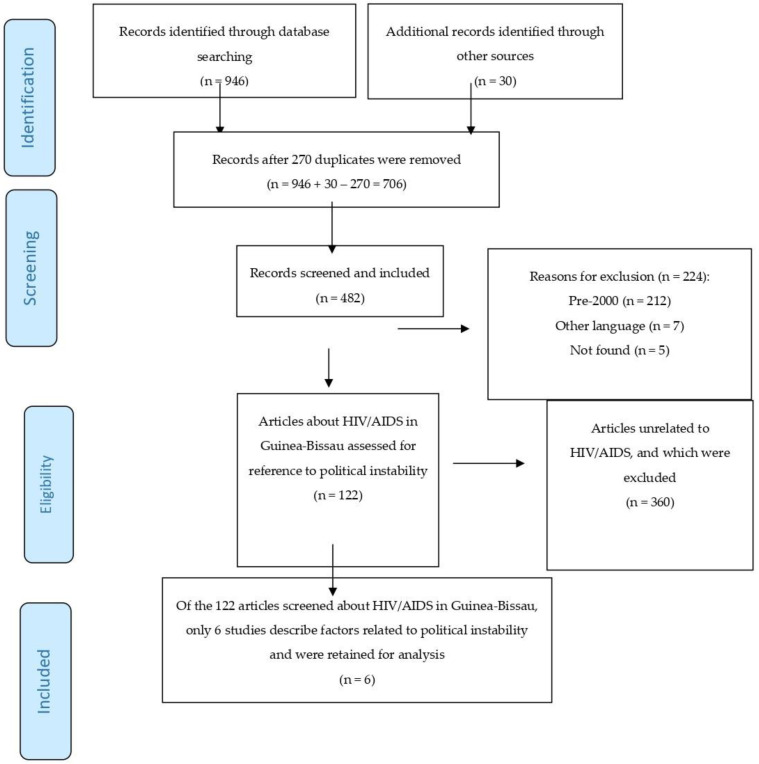
PRISMA flowchart.

**Figure 2:**
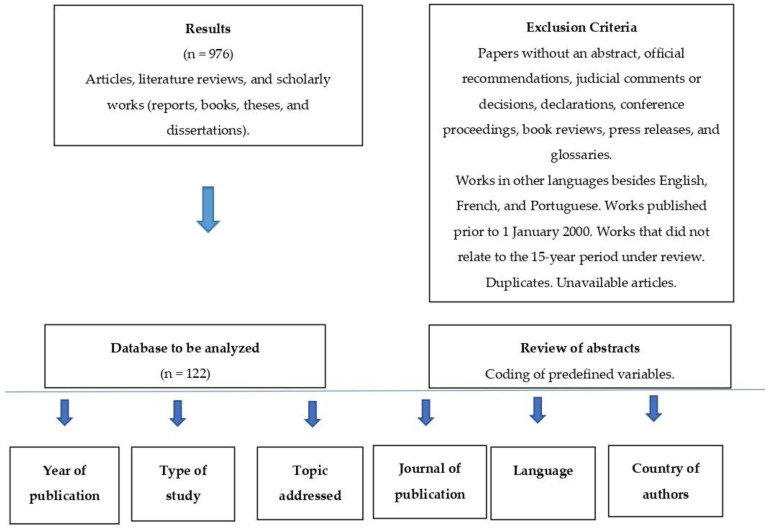
Methodology of the research strategy.

**Figure 3:**
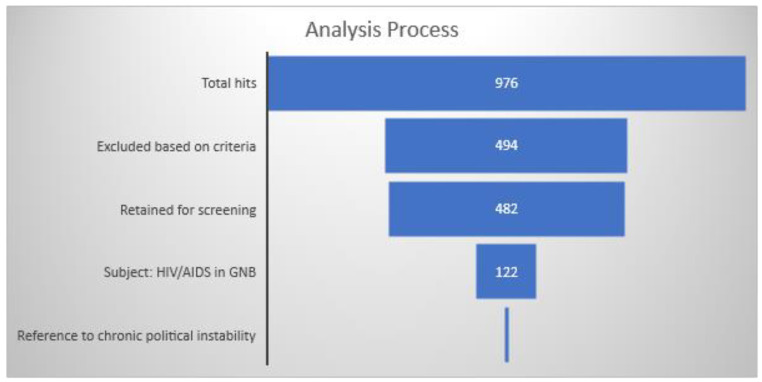
Results of analysis

Studies were screened by the first author (J.G.). Titles and abstracts were reviewed. The last author (E.M) reviewed the work, and where there was disagreement, it was presented to a third co-author until unanimity was reached. Duplicate articles were eliminated. A data collection form in Excel included the following information for each study meeting the inclusion criteria: year of publication, journal, type of study, research topic, country affiliation of first author, and language. The consensus-based database of unique articles was constructed and reviewed by J.G. and E.M. for potential coding errors. The systematic review was conducted following the PRISMA guidelines. Figure 1 presents the PRISMA flow chart, Figure 2 summarizes the methodology of the research strategy, and Figure 3 illustrates the results of the analysis process.

## 3. Results 

**Figure 4:**
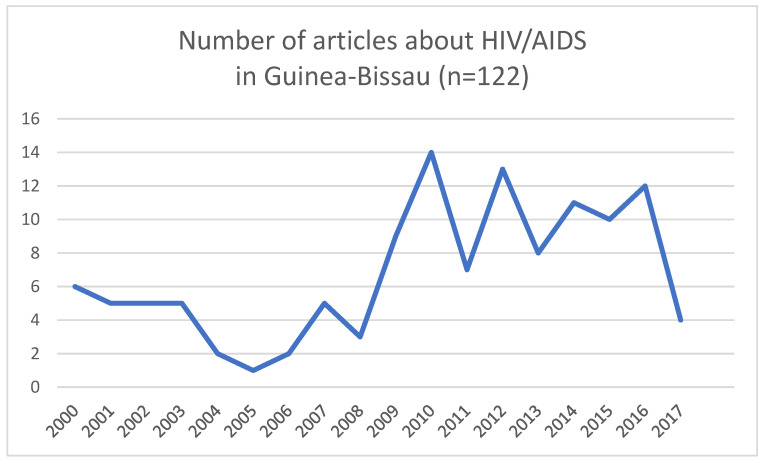
Year of publication (n = 122).

The search yielded 946 records from search engines, and an additional 30 articles from the grey literature were added. Exclusion criteria were applied, and the following records were eliminated: duplicates (270), articles published prior to the year 2000 (212), articles written in other languages (7), and articles not found (5). We screened the remaining 482 articles for eligibility and identified 122 articles about HIV/AIDS in Guinea-Bissau during the 15-year period under review. The results are summarized in Table 1, Table 2, Table 3 and Table 4. From 2010 to 2017, there was generally an increase in the number of articles published about HIV/AIDS in Guinea-Bissau compared to the period of 2000–2009. Between 4 and 14 articles were published annually from 2010 to 2017 (see Figure 4). In total, we found 36 studies based on cohort analyses (29.5%) of which 6.6% were specified as prospective analyses, 6.6% were specified as retrospective (6.6%) analyses, and the remainder unspecified (16.4%). Studies based on clinical microbiology (20.5%) were next represented, followed by cross-sectional analyses (13.1%). 

We conducted a textual analysis of the 122 articles about HIV/AIDS in Guinea-Bissau (Figure 4). The 122 retained articles were reviewed for any reference to themes related to political instability, e.g., assassinations, coup d’états, governance, and civil war or any of its variants. Six articles described factors related to political instability, and they are summarized in Table 4 The six studies were biomedical in nature and published in biomedical journals. Study populations comprised HIV or TB patient populations, pregnant women, military personnel, and police officers. There were no standard measurements used to measure the effects of political instability.

Four articles described the effects of the civil war (1998–1999) on their respective study populations, measuring effects before, during, or after the civil war [11,12,13,14]. The four studies measured the effects of the civil war on an ongoing cohort being monitored prior to the start of the war. The study of Biague [15] looked at increasing trends in HIV prevalence among military personnel but did not situate his study into a pre- and post-war design; the author offered evidence of high sexual risk-taking but did not explore the effects of the civil war or subsequent years of political instability. 

Given the relevance and importance, one article published in 2018 was intentionally added to this research [16]. Of all the studies reviewed, the study of Rasmussen et al. was the most relevant because it most closely responded to our research question, being the only article that included political instability in the title while also framing analysis with reference to health system resilience. This article examined the effects of periods of political instability on public health outcomes by establishing an association between periods of political instability and periods of decreased HIV testing among pregnant women at the national reference maternity hospital. 

## 4. Discussion

Biomedical, clinical, or epidemiological research dominated public health research production on HIV/AIDS in Guinea-Bissau during the MDG period. We identified a total of 122 articles meeting inclusion criteria. We noted an increase in the number of articles published in the period of 2010–2017 when compared to 2000–2009. This could be linked to the increase in the number of open access journals that have become more common in the last 15–20 years, increased funding for research, and greater attention and donor support to the global HIV/AIDS epidemic. We found six that considered the effects of political instability in their study design. Studies focusing on HIV-2 predominated (43.4%), followed by microbiology (9.0%) and opportunistic infections (8.2%). The large proportion of studies related to HIV-2 may be unsurprising given that the origins of HIV-2 have been traced to Guinea-Bissau and the country has the world’s highest HIV-2 prevalence [17]. 

Our review focused on the country’s post-civil war period and should be contextualized in the country’s larger post-independence history. The economic, political, and public health situation of Guinea-Bissau was arguably already worse than its neighbors prior to 2000, which likely contributed to the country’s worse HIV/AIDS indicators during the MDG period. Our search found four articles examining the effects of the civil war on HIV/AIDS services. The civil war of 1998–99 had a profound effect by exacerbating brain drain, increasing outward migration, and leading to a general breakdown of public administration and infrastructure. Four studies looked at the effects of the civil war on HIV/AIDS outcomes. There was sparse literature, however, on any effects of the ensuing 15 years of post-war chronic political instability. 

Guinea-Bissau stands out as a nation that has struggled to establish a stable post-independence political order and remains one of the world’s poorest, least developed, and most fragile countries. Its chronic political instability has stymied its economic and social development, and it continues to affect the daily lives of its citizens [18,19]. Our study raises questions about the nature of research conducted on HIV/AIDS in Guinea-Bissau. Given the fragility of Bissau-Guinean society in the post-civil war period (exacerbated by the chronicity of its cycles of political instability), we expected a larger share of the literature to explore the social and political determinants of HIV/AIDS. Instead, we found a literature that followed, with few exceptions, a nearly monolithically biomedical approach that appears to have largely ignored political instability in the country other than a reference to the 1998–1999 war. What we found missing from the literature is an explicit link between the risks of HIV/AIDS infection and the vulnerabilities created by social forces and likely worsened by political instability, such as poverty, gender-based violence, the mismanagement of public resources, and a lack of employment opportunities, to name a few. 

The protracted nature of Guinea-Bissau’s instability has had pernicious effects on the social sectors, including health [20]. The human resources for health crisis has been described [21], as have the effects of instability on the country’s research agenda [22]. These effects have similarly been assessed with regard to health planning in the country since the first National Health Development Plan (PNDS) was introduced in 1997, pointing towards the negative impact of political instability and state fragility to the extent of hindering the implementation of the first PNDS [23]. 

Given that Guinea-Bissau is one of the most politically unstable countries in the world, our findings of mostly biomedical research performed to date on the HIV/AIDS epidemic in Guinea-Bissau might be considered surprising. While the multidisciplinary field of political epidemiology has burgeoned since the 1980s, it is, by no means yet a mainstream approach integrated into public health perspectives [24]. A recent review of systematic reviews on the impact of political economy on population health highlighted gaps in the literature and called for better quality reviews [25]. Indeed, the importance of developing an explicit political epidemiology of HIV and translating it into public health research has been emphasized [26]. The conducive environment that political instability creates for HIV/AIDS in the sub-Saharan African setting has been described [27], and links between income, HIV/AIDS, and political instability as predictors of mortality have been established [28]. An important challenge comes from the fact that the effects of political instability on health outcomes are not easily quantitatively measured, standard frameworks and measurement tools are missing, and the potential for mutual learning between the fields of political science and public health has not yet been fully unlocked [29,30,31].

Political epidemiology may serve as a useful tool to generate approaches and frameworks for systematically assessing political barriers and facilitators to HIV/AIDS responses. Gil-González et al. proposed a research agenda for how political epidemiology can help to address why the MDGs were not met [24]. Their proposal included a focus on “research issues relating to aspects that have not yet been analyzed or have been insufficiently investigated with respect to MDG,” and these included “war, political repression or structural violence, population displacement or forced migration; the human rights situation.” Beyond the realm of HIV/AIDS, more and better quality tools, methods, and frameworks as part of an overall political epidemiological research agenda is still needed. Writing about the political epidemiology of disease eradication programs, Taylor noted the limited extent to which socio-political factors are considered in program conceptualization, design, and implementation [32]. Mixed method work combining qualitative and quantitative approaches may be a step in the right direction, e.g., Martins et al.’s work on the challenges facing a malaria control program during a period of political instability in Timor-Leste [33] or Ruckstuhl et al.’s effort to use malaria surveillance data collected by community health workers from the Central African Republic to document barriers to access during a civil war [34]. 

A limitation of this study relates to the scope of the research question—widening the question beyond HIV/AIDS may have yielded additional hits. The small number of hits with our search criteria confirmed the relevance of this review in pointing at key lacuna in HIV-related research in Guinea-Bissau that should be addressed. 

The search criteria focused on the effects of chronic political instability on a single health outcome (HIV/AIDS) rather than on another disease outcome or on the health sector more broadly. We chose to limit our search to HIV/AIDS because Guinea-Bissau is disproportionately affected by the disease among other countries in the West Africa region, as well as for the pragmatic purpose of rendering our systematic review more manageable. A second limitation may be a risk of publication bias, given the limited number of relevant studies found in the literature that met the search criteria and the fact that work conducted on this topic may not have been submitted for publication. 

Future research could aim to illuminate the effects of chronic political instability on HIV/AIDS outcomes and local strategies for resilience and adaptation. Qualitative research could explore the following research domains: institutional effects (the effects of weak governance on the institutions that are charged with coordinating and implementing the HIV/AIDS response); economic, social, and human rights effects (the role of political instability in intensifying vulnerability); behavioral effects (effects of crisis, conflict, or instability in increased risk of HIV acquisition); decentralized effects (effects of instability at lower levels of the health system); epidemiological effects (considering whether there a discernible time-series link between trends in the distribution of disease and political events); financial effects (consequences of large funding needs in the absence of adequate government contributions to the health sector and, more specifically, the HIV/AIDS response); partnership effects (the effect on the creation of new partnerships and the mobilization of external resources); and the culture of violence (how the country’s history has inculcated a culture of violence conducive to the spread of HIV/AIDS, e.g., through gender-based violence). 

## 5. Conclusions

Guinea-Bissau has experienced a high burden of HIV/AIDS and cycles of political instability that have likely hampered its HIV/AIDS response. We identified scarce results exploring how its post-civil war political instability may have contributed to its high HIV/AIDS disease burden. Our review suggests that most of the research on this “outlier” epidemic in West Africa has followed a predominantly biomedical paradigm. Future research on the HIV/AIDS epidemic in Guinea-Bissau should apply a political epidemiology approach that considers the social and political determinants of HIV/AIDS and recommends how the HIV/AIDS response can be strengthened in light of the country’s political instability. 

## Figures and Tables

**Table 1:** Most common study types found (n = 122).

Study Type	n	%
Clinical microbiology	25	20.5%
Cohort	20	16.4%
Cross-sectional	16	13.1%
Prospective cohort	8	6.6%
Retrospective cohort	8	6.6%
Case report	6	4.9%
Molecular epidemiology	4	3.3%
Editorial	3	2.5%
Qualitative	3	2.5%
Comment	3	2.5%
Screening	2	1.6%
Case control	2	1.6%
Evaluation	2	1.6%
Historical	2	1.6%
Modelling	2	1.6%
Not stated	2	1.6%
Prospective follow up	2	1.6%
Vaccine research	2	1.6%
Clinical trial	1	0.8%
Longitudinal study	1	0.8%
Narrative	1	0.8%
Observational	1	0.8%
Prevalence	1	0.8%
Retrospective cross-sectional	1	0.8%
Sentinel surveillance	1	0.8%
Seroprevalence	1	0.8%
Test comparison	1	0.8%
Testing	1	0.8%
	122	100%

**Table 2:** HIV/AIDS sub-topics (n = 122).

HIV/AIDS Sub-Topic	n	%
HIV-2	53	43.4%
Microbiology	11	9.0%
Opportunistic infections	10	8.2%
TB/HIV	7	5.7%
Human T-lymphotropic virus (HTLV)	6	4.9%
Prevalence	6	4.9%
Vaccine	6	4.9%
Antiretroviral therapy (ART)	5	4.1%
Lost to follow up	4	3.3%
Testing	4	3.3%
Prevention of mother to child transmission	3	2.5%
Male circumcision	2	1.6%
Diabetes	1	0.8%
Diarrhea	1	0.8%
Drug resistance	1	0.8%
Mortality	1	0.8%
Orphans	1	0.8%
	122	100%

**Table 3:** Country affiliation of first author (n = 122).

Country of First Author	n	%
Guinea-Bissau	42	34.4%
Sweden	21	17.2%
The Gambia	14	11.5%
Denmark	10	8.2%
U.K.	10	8.2%
Portugal	7	5.7%
Not indicated	6	4.9%
Italy	4	3.3%
Canada	2	1.6%
U.S.A.	2	1.6%
Brazil	1	0.8%
India	1	0.8%
Senegal	1	0.8%
Switzerland	1	0.8%
	122	100%

**Table 4:** Studies exploring relation between political instability and HIV/AIDS in Guinea-Bissau during the period of 2000–2015 in chronological order (n = 6).

Author and Year	Study Design	Objective	Population	Result	Potential Effect of Political Instability
Gustafson, 2001 [11]	Retrospective cohort study	“To determine the effectof irregular TB treatment due to an armed conflict”.	320 TB patients	“Mortality rate ratio was 3.12 in the war cohort” and “each additional week of treatment before the war started increased probability of survival by 5%.”	“Interruption of treatment had a profound impact on mortality“Regular treatment for TB was associated with significantly improved survival for HIV-infected patients.”
Månsson, 2007 [12]	Consecutive sampling of data from the sentinel surveillance program	“To examine… trends of HIV prevalence from antenatal surveys from 1987 to 2004” and determine if the civil war affected HIV prevalence.	20,422 pregnant women	“HIV-1 prevalence increased from 0.0% in 1987 to 4.8% in 2004 and…HIV-2 decreased from 8.3% to 2.5”	Civil war may have ledto HIV-1 prevalence doubling from 1997 to 1999, but “no evidence of a long-term effect on the trends on HIV-1 or HIV-2 prevalence.”
Gustafson, 2007 [13]	Risk factor assessment during 8-month TB treatment	“To assess easily monitored predictorsfor TB mortality”.	440 male and 269 female TB patients	“Case fatality rates for HIV-positive [TB patients] were higher during (35%) and after the war (29%) compared to before the war (17%)”.	Case fatality rates for co-infected patients “were higher during (35%) and after the war (29%) compared to before the war (17%)”.
Månsson, 2009 [14]	Open prospective cohort study	“To study prevalence and incidence of HIV-1 and HIV-2 between 1990 to 2007 and to examine impact of the civil war (1998–1999)”	4592 police officers	“HIV-1 prevalence increased… from 0.6% to 3.6% before the war and was 9.5% in the first serosurvey after the war. HIV-1 incidence more than doubled during and shortly after the war, from 0.50 to 1.22 per 100 person-years…HIV-2 prevalence decreased from 13.4 to 6.2% during the entire study period.”	“The civil war…appears to have induced a temporary increase in HIV-1 transmission, but now a stabilization of HIV-1 incidence and prevalence seems to have taken place.”
Biague, 2010 [15]	Repeated cross-sectional HIV-1 and HIV-2 surveys	“To determine HIV prevalence, trends, and risk factors in the military”.	2317 military personnel	Increasing trends in HIV-1 infection (1.1% in 1992–1995 to 7.7% in 2005) among military personnel.	“Increasing trend of HIV-1 and...high risky sexual behavior…among military personnel”.
Rasmussen, 2018 [16]	Retrospective cross-sectional study	To assess HIV testing among pregnant women and factors associated with non-testing.	31,443 pregnant women presenting for delivery	“Opt-out HIV testing at labor increased from 38.1% (2008) to 95.7% (2013) There were four distinct periods (two or more consecutive calendar months) when less than 50% of women delivering were tested”.	“Periods of political instability were significantly associated with not testing for HIV”.

## Data Availability

Not applicable.

## Appendix A

**Table A1:** Cross-country comparison of HIV/AIDS incidence and prevalence in Guinea-Bissau, The Gambia, Senegal, and Guinea (2019).

Country	Guinea-Bissau	The Gambia	Senegal	Guinea
HIV incidence per 1000 people (adults 15–49)	1.85 [1.47–2.31]	1.77 [1.17–2.73]	0.14 [0.10–0.20]	0.68 [0.47–0.96]
HIV incidence per 1000 people (all ages)	1.15 [0.92–1.41]	1.06 [0.70–1.62]	0.09 [0.07–0.13]	0.39 [0.28–0.54]
Adults aged 15–29 prevalence rate	3.4 [2.8–3.9]	1.9 [1.5–2.4]	0.4 [0.3–0.4]	1.4 [1.2–1.6]
Women aged 15–29 prevalence rate	4.1 [3.5–4.7]	2.3 [1.8–2.9]	0.4 [0.4–0.5]	1.8 [1.5–2.1]
Men aged 15–29 prevalence rate	2.6 [1.9–3.0]	1.5 [1.1–1.9]	0.2 [0.3–0.4]	0.9 [0.7–1.1]

Source: UNAIDS Country Fact Sheets https://www.unaids.org/en/regionscountries/countries/guinea-bissau accessed on 11 July 2020.

## Appendix B

**Table A2:** Major milestones of political instability in Guinea-Bissau during the Millennium Development Goal (MDG) era.

Month/Year	Political Event in Guinea-Bissau
June 1998–May 1999	Civil war
January 2000	“Kumba Yala elected president”
November 2000	Assassination of General Mane following alleged coup attempt
December 2001	Announcement by government that it has foiled an attempted coup by the army. Dismissal of Prime Minister Faustino Imbali
September 2003	Coup d’état led by the military pushes out President Kumba Yala
July 2005	“Joao Bernardo Vieira wins presidential elections”
March–April 2007	“Prime Minister Aristides Gomes resigns”
August 2008	“President Vieira dissolves parliament”
November 2008	Attack on President Vieira’s house by soldiers. He survives the failed coup attempt.
March 2009	A bomb explosion kills the chief of staff of the army, General Tagme Na Waie. A short while later, President Vieira is assassinated by soldiers.
July 2009	“Malam Bacai Sanha wins presidential election”
December 2011	Announcement by Prime Minister Carlos Gomes Junior that an attempted coup against President Malam Bacai Sanha was prevented
January 2012	Death of President Malam Bacai Sanha in France
April 2012	Coup d’état leads to the arrest of interim President Raimundo Pereira and former Prime Minister Carlos Gomes Junior. An internationally unrecognized, transitional government takes power.
October 2012	Unrest in the barracks of the armed services leads to the death of seven people. “The transitional government describes [it] as a foiled coup attempt.”
May 2014	The country holds what many outsiders consider to be free and fair elections. Jose Mario Vaz is elected President.
May 2015	At a donor roundtable held in Brussels, approximately USD 1.1 billion is pledged by the international community for Guinea-Bissau’s development “after years of instability”
August 2015	President Jose Mario Vaz dismisses the Prime Minister Domingos Simões Pereira which creates a crisis in the government that would last until the end of his mandate in 2019

Source: Based on and adapted from Guinea-Bissau profile—Timeline. BBC News. https://www.bbc.com/news/world-africa-13579838 accessed on 11 July 2020.
